# Vangunu giant rat (*Uromys vika*) survives in the Zaira Community Resource Management Area, Solomon Islands

**DOI:** 10.1002/ece3.10703

**Published:** 2023-11-20

**Authors:** Tyrone H. Lavery, Adrian Holland, Nixon Jino, Atuna Judge, Hikuna Judge, Pandakai Onga, Kevin Sese

**Affiliations:** ^1^ School of BioSciences The University of Melbourne Melbourne Victoria Australia; ^2^ Zaira Rangers Zaira Village Western Province Solomon Islands; ^3^ School of Natural Resources and Applied Sciences Solomon Islands National University Honiara Solomon Islands

**Keywords:** conservation, deforestation, indigenous protected area, mammal, survey

## Abstract

Described in 2017 and known only from the holotype, *Uromys vika* is surely among the world's least studied rodents. This critically endangered species is facing a rapidly increasing scale for threat from logging of its primary lowland forest habitat, on the only island on which it occurs—Vangunu, Solomon Islands. However, a deep traditional ecological knowledge of *U. vika* is held by Vangunu's people. Using camera traps and guided by this knowledge, we aimed to make additional records of *U. vika* in the last major block of Vangunu's primary forest. We successfully captured 95 images of what we postulate is four different individuals. The forests at Zaira represent the last suitable habitat remaining for this species, and recent development consent for logging at Zaira will lead to its extinction if permitted to proceed.

## Graphical abstract

Described in 2017 and known only from the holotype, *Uromys vika* is one of the world's most data‐poor rodent species. However, a deep traditional ecological knowledge of *U. vika* is held by Vangunu's people. Using camera traps and guided by this knowledge, we successfully captured 95 images of what we postulate is four different individuals.

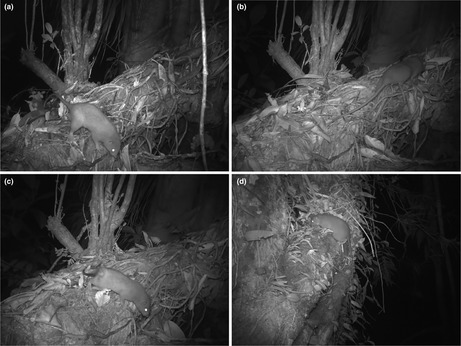

## 1 INTRODUCTION

The Solomon Islands archipelago lies in a global biodiversity hotspot (Mittermeier et al., 2004). The major islands are home to extraordinary examples of insular evolution (Dutson, 2011; Lavery & Flannery, 2023; McCoy, 2006; Pikacha et al., 2008). However, substantial Wallacean and Linnaean shortfalls remain among the vertebrate faunas, and the deep Indigenous knowledge that persists widely today is a key tool to reduce these (e.g. Alabai et al., 2019; DeCicco et al., 2019, 2020; Jenkins et al., 2008; Lavery et al., 2021; Lavery, Alabai, et al., 2020; Lavery, Posala, et al., 2020; Pollard et al., 2015). People from southern Vangunu continue to hold intimate knowledge of a native species of giant rat known by the Indigenous language name *vika*. For decades, anthropologists and mammalogists alike were aware of this knowledge (see Fisher & Tasker, 1997; Hviding, 2005 for examples). Nevertheless, periodic efforts to scientifically identify and document this species were fruitless (e.g. Fisher & Tasker, 1997). Likewise, more intensive surveys from 2010 to 2015 using camera traps, aluminium box traps, spotlighting, and active searches of hollow trees failed to confirm the existence of *vika*. Instead, the only rodent species documented was the pervasive introduced black rat (*Rattus rattus*) (Lavery & Judge, 2017).

Ironically, it was a commercial logging company operating in southern Vangunu's primary forests that finally produced the vital evidence that was needed. The felling of a large habitat tree (*Dillenia salomonensis*) fatally injured one of the rodents that must have been sheltering somewhere in its canopy or hollows. Partial remains accessioned to the collections of the Queensland Museum, Brisbane, Australia, were sufficient for comparisons with described rodents of northern Melanesia, and it was subsequently described as a new species *Uromys vika* (Lavery & Judge, 2017).

The Vangunu giant rat was the first new species of murid described from Solomon Islands in over 80 years. This critically endangered rodent (Lavery, 2019) is endemic to Vangunu Island, Western Province, and all evidence has indicated it can only persist in primary lowland forests (Lavery & Flannery, 2023; Lavery & Judge, 2017). This habitat is rapidly declining on Vangunu due to commercial logging, and the area in which the only known specimen was found has now been comprehensively logged, rendering it unsuitable for *U. vika* (Hansen et al., 2013; Lavery, Posala, et al., 2020). Lavery and Judge (2017) postulated the largest remaining tract of Vangunu's primary forests (recognised as the Zaira Community Resource Management Area–herein referred to as Zaira) was probably the last remaining habitat for *U. vika*. The community at Zaira was adamant the species lived in their forests. However, the presence of *U. vika* had never been scientifically documented there. Here, we aimed to confirm the persistence of *U. vika* on Vangunu Island by documenting it at Zaira via the use of camera traps.

## 2 METHODS

**FIGURE 1:**
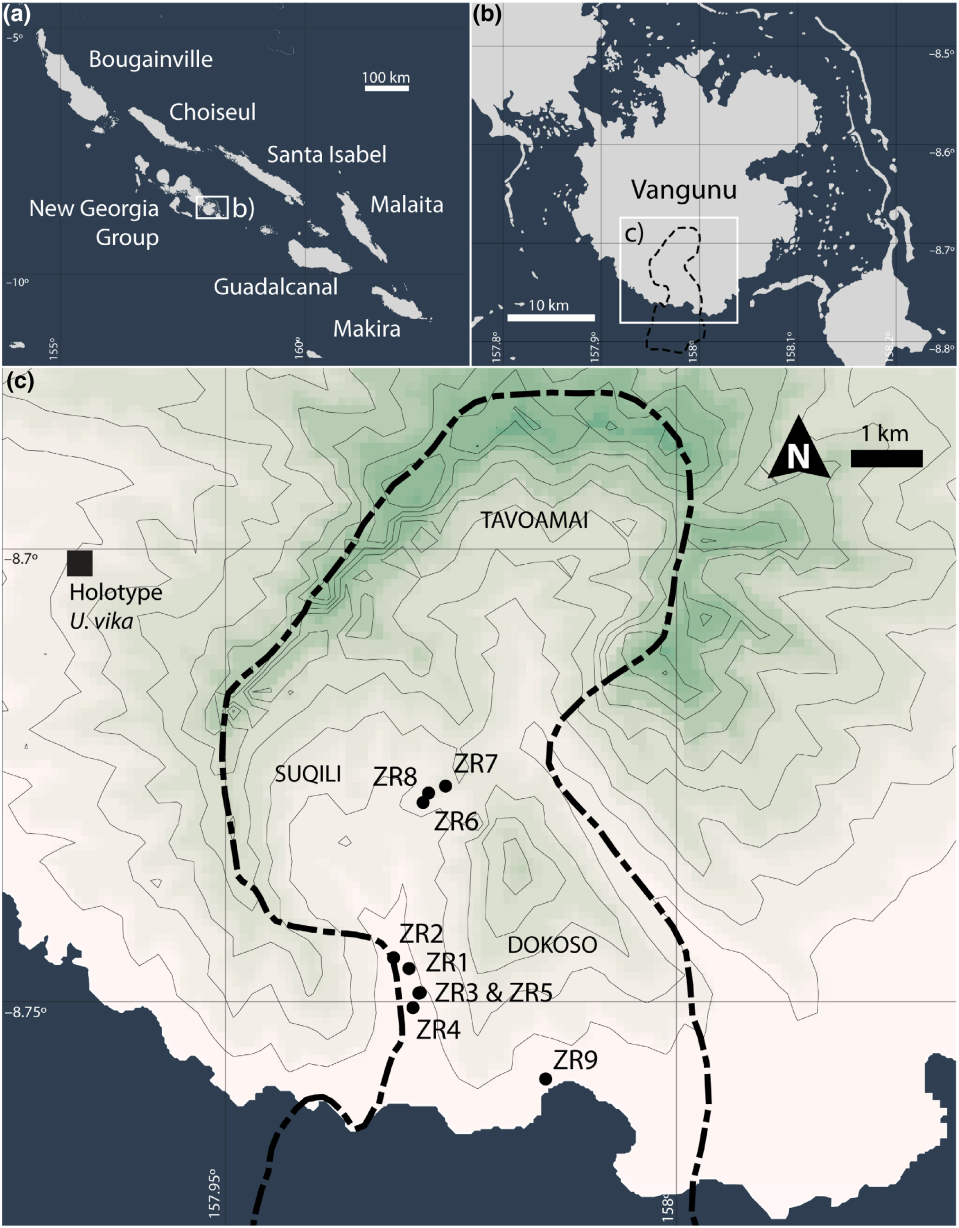
Location of (a) Vangunu Island, Solomon Islands; (b) location of the Zaira Community Resource Management Area (ZCRMA) outlined in the black dotted line; and (c) the locations of camera traps deployed to survey *Uromys vika*, dark lines are geographic coordinates (linear) or 100 metre contours (irregular lines).

The Zaira Conservation Resource Management Area comprises approximately 60 km^2^ of terrestrial and marine environments. The terrestrial component is made up of three tribal areas–Dokoso, Tavoamai, and Suqili (Figure 1). These terrestrial lands comprise a total area of 36 km^2^, forming a continuous block of primary forest from sea level to the rim of the Vangunu stratovolcano over 1000 metres in elevation.

Between September 2020 and August 2021, we surveyed mammals using nine camera traps (Reconyx PC850 & PC900). Eight were placed in arboreal positions (10–20 m above the ground) in areas of lowland primary forests (below 400 m elevation) (Figure 1, Table 1). A ninth camera was placed in a position on the forest floor. Camera triggers were set to high sensitivity, 5 pictures per trigger with rapidfire intervals, and no delay between triggers. A lure was placed on a branch 1–2 m in front of the camera and secured to vines and epiphytes with wire. The lure consisted of a small glass oil lamp with a cotton wick, filled with commercially available sesame oil. This provided a scent attractant that we intended would last for months in duration and could not be consumed by wildlife.

**TABLE 1:** Site elevation, height of camera placement above the ground, and tree genus chosen for the placement of nine camera traps targeting *U. vika* in the Zaira Community Resource Management Area.

Site	Elevation (m)	Height (m)	Tree genus
ZR1	67.9	15.9	*Dillenia*
ZR2	71	0.5	*Canarium*
ZR3	70	10	*Canarium*
ZR4	73	17	*Dillenia*
ZR5	70	23	*Dillenia*
ZR6	242	12	*Canarium*
ZR7	266	10	*Calophyllum*
ZR8	257	16.5	*Dillenia*
ZR9	0	16	*Vitex*

*Note*: Locations are illustrated in Figure 1.

Camera sites were selected by members of the study team from Zaira Village (AH, NJ, AJ, HJ, and PO) using their traditional knowledge of *U. vika* and three habitat criteria. First, we targeted trees adjacent to a Solomon Islands endemic nut tree known locally as a preferred food species for *U. vika* (*Canarium salomonense*). Second, the trees chosen were large with dense growths of bryophytes, and epiphytic plants such as ferns. According to Zaira's residents, these conditions provide suitable nesting places for *U. vika*. Third, we placed cameras in areas where activity of *U. vika* was evidenced by the occurrence of matured *C. salomonense* nuts on the ground with large incisor gnaw marks.

We examined camera trap data using the packages “circular” (Agostinelli & Lund, 2022) and “overlap” (Ridout & Linkie, 2009) in R (R Core Team, 2022). We treated consecutive images of the same species recorded on the same camera as independent observations if they were separated by a time period of 30 min or more. We calculated overlap coefficients of temporal activity patterns (Dhat1) for *U. vika* and other arboreal mammals, this is a descriptive measure with no associated test for statistical significance.

## 3 RESULTS

**FIGURE 2:**
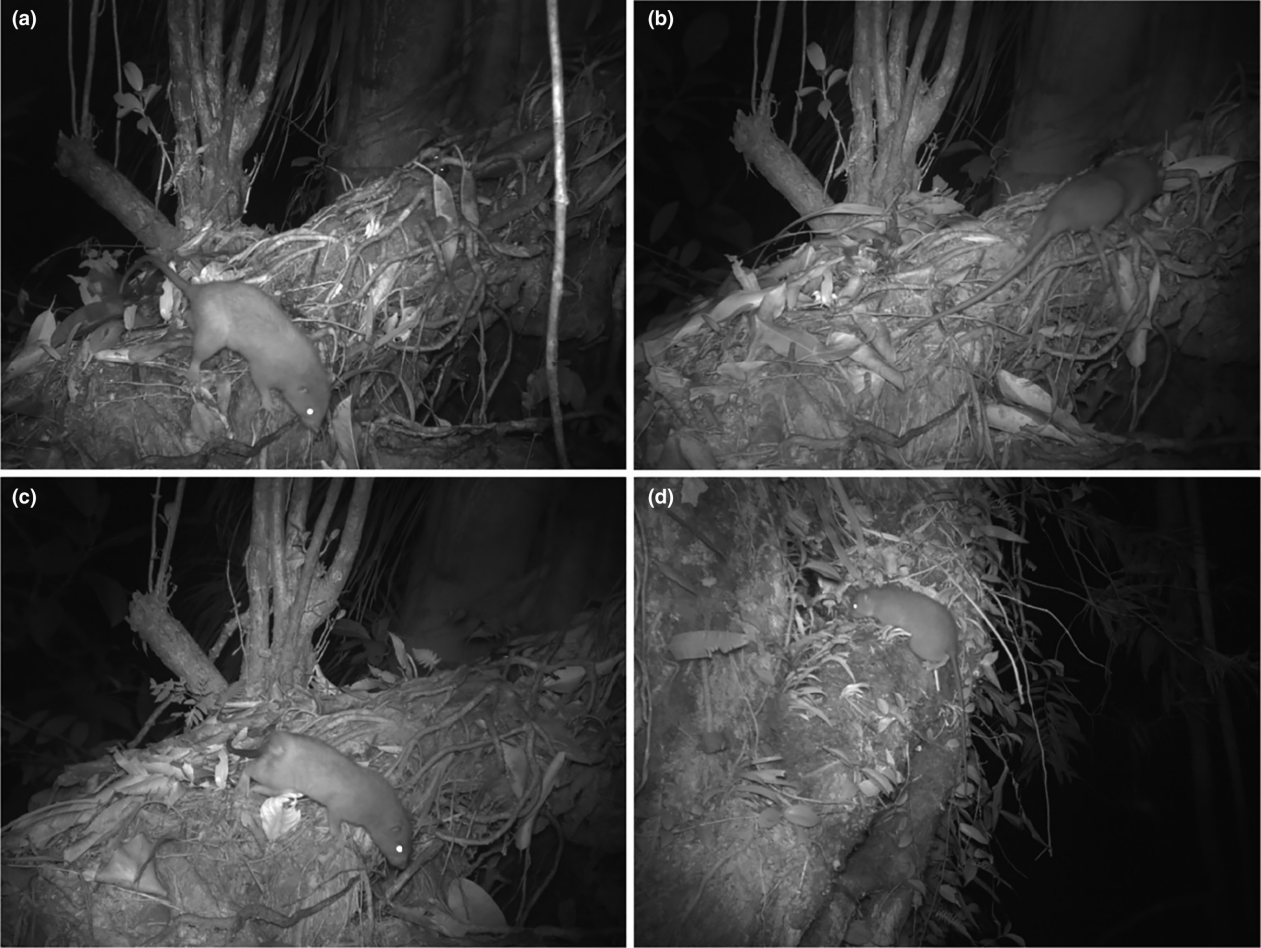
Examples of camera trap images of *Uromys vika* recorded during this study in the Zaira Community Resource Management Area, Vangunu Island, Solomon Islands: (a) male at site ZR5; (b) female at site ZR5; (c) second female with scar on rump at site ZR5; and (d) female at site ZR9.

We successfully captured images of *U. vika* at two of our Zaira survey sites in the Dokoso and Suqili tribal areas (cameras ZR5 and ZR9, Figure 1). The rodents were irrefutably identified as *U. vika* by their large body size, long tails, and the presence of very short ears (see examples in Figure 2a–d). A total of 95 images containing *U. vika* were recorded. On camera ZR5, we recoded images of multiple individuals. One was a male, identified by the presence of large testes (Figure 2a). A second individual was identified as a female by the absence of testes (Figure 2b), and the third was apparently a second female, discernible from the first female by the presence of a major scar on the right side of the rump (absent in the first female and the male) (Figure 2c). On the second camera (ZR9), we recorded just a single individual, that we also deemed to be a female due to the lack of obvious testes (Figure 2d). These two camera sites were separated by a straight‐line distance of approximately 2 km and we suggest the single individual on camera ZR9 is thus likely an additional individual not among the three recorded on camera ZR5. Up to four individuals were thus identified in the Zaira Community Resource Management Area.

**FIGURE 3:**
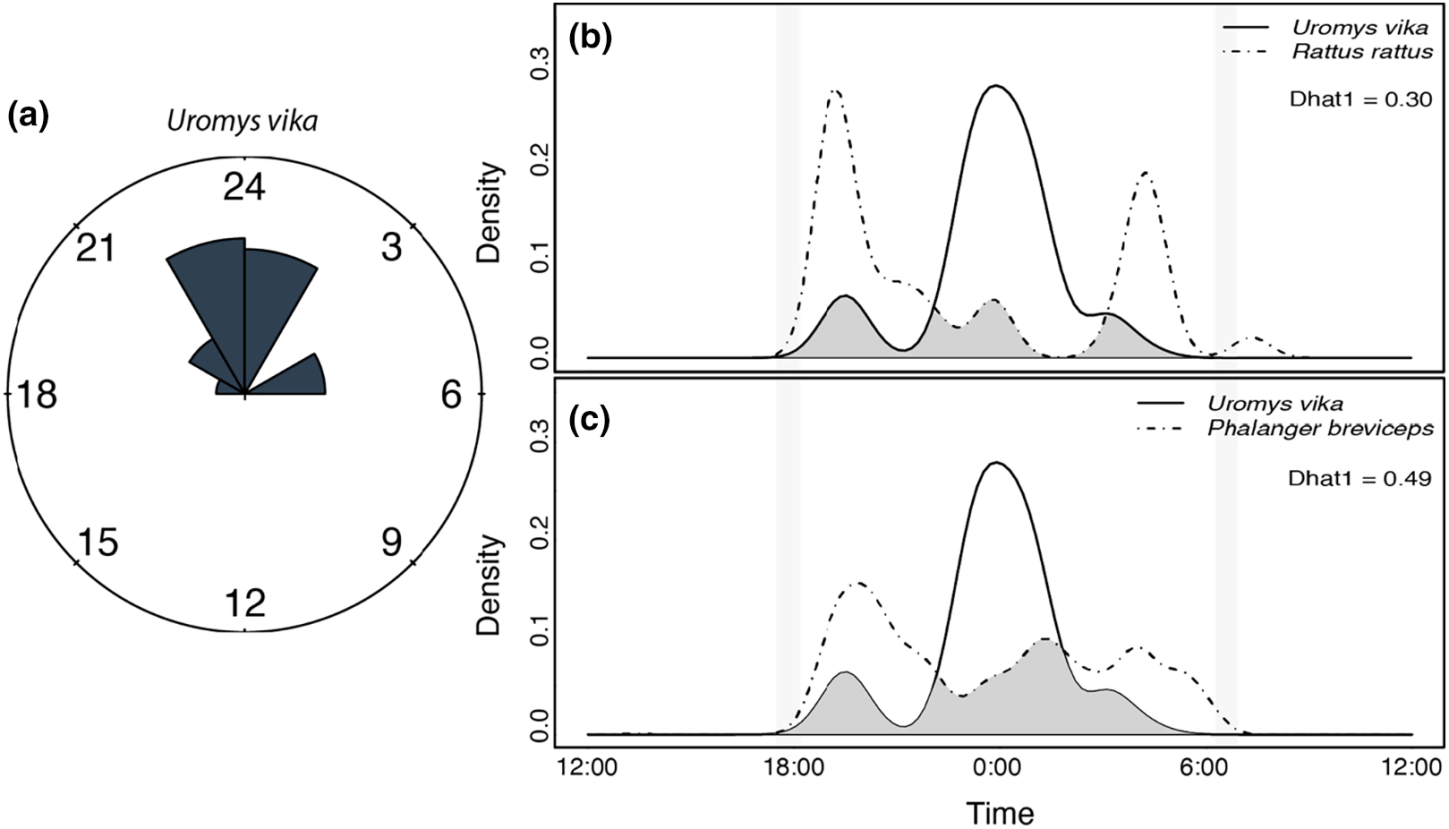
Activity patterns of mammals detected on camera traps in the Zaira Community Resource Management Area, Vangunu Island, Solomon Islands: (a) temporal patterns in detection of *Uromys vika*; (b) comparisons of temporal overlaps in *Uromys vika* and *Rattus rattus* detections; (c) comparisons of temporal overlaps in *Uromys vika* and *Phalanger breviceps* detections. Gray vertical bars indicate approximate times of sunrise and sunset at the study site.

Other species documented during the survey period were Bismarck common cuscus (*Phalanger breviceps*), black rat (*Rattus rattus*), buff‐headed coucal (*Centropus milo*), mangrove monitor (*Varanus indicus*), and brahminy kite (*Haliastur indus*). Detections of *U. vika* were centered on midnight (Figure 3a), while detections of *R. rattus* and *P. breviceps* were clustered around the hours following sunset and prior to sunrise. Temporal overlaps between *U. vika* and *R. rattus* or *P. breviceps* were 0.30 and 0.49, respectively (Dhat1) (Figure 3b,c). No direct interactions between *U. vika* and other species were recorded on camera, and no two *U. vika* individuals were recorded in the same image.

## 4 DISCUSSION

Our results reported here represent the only known records of *U. vika* other than the holotype. Confirmation that *U. vika* remains extant on Vangunu Island, in the last existing major tract of primary forest, is thus extremely positive news for this poorly known species. The result that our camera traps recorded up to four individuals also is encouraging. Beyond confirming presence of *U. vika*, we are unable to offer major advances in the knowledge of the rodent's ecology or conservation needs, and our data are insufficient to identify seasonal patterns in behaviour or detectability. We recorded *U. vika* in tree species belonging to the genera *Calophyllum* and *Dillenia*, the holotype was captured from *Dillenia salomonensis* (Lavery & Judge, 2017), and all records have now been from primary forests below an elevation of approximately 250 m. Unfortunately, species of *Dillenia* are preferentially targeted by commercial logging companies in Solomon Islands, and commercial logging is legally restricted to elevations below 400 m. All images were captured during nocturnal hours, and activity was clustered around midnight. Comparisons with the temporal activity patterns of other species indicated there could be avoidance of interactions with *R. rattus* and *P. breviceps* by *U. vika*, but the data are sparse and more work is needed to confirm this.

Our choice of lure may represent an important advancement in efforts to document endemic Solomon Islands rodents with camera traps. Previous attempts at the same site (using just three camera traps) used lures made of peanut butter placed on fibre wadding inside PVC cannisters. These cameras only recorded the introduced *R. rattus*. The peanut butter was also heavily degraded by the time cameras were recovered after 2 months and probably no longer acted as an attractant for rodents. Glass oil lamps filled with sesame oil proved successful at attracting *U. vika*. Ten months after deployment of the cameras, we still detected *U. vika*, and the individual recorded was clearly investigating the lure. Both trees in which *U. vika* were recorded were identified as being suitable survey sites because they were adjacent to ngali nut trees (*Canarium salomonense*). Oil derived from *Canarium* nuts (*C. indicum*) is commercially available in Solomon Islands, and we suggest this may prove an even more successful lure in future survey trials for this and other Solomon Islands rodents. This native nut tree when in fruit is a powerful attractant for Bougainville Giant Rat (*Solomys salebrosus*) (Lavery & Flannery, 2023). However, commercially available *Canarium* oil could not be sourced for this survey.

The results presented here come at a critical juncture for the future of the Zaira Community Resource Management Area. The residents of Zaira Village for the past 16 years have pursued their desire to protect the Dokoso, Tavoamai, and Suqili tribal areas from commercial logging. Many attempts and efforts have been made to register these lands as a protected area under the *Solomon Islands Protected Areas Act 2010*. Nonetheless, on November 1, 2022, the Solomon Islands Government granted development consent for commercial logging of Dokoso customary land. Zaira's representatives from Dokoso tribe have lodged an appeal against this decision and the result will be critical for the conservation status of *U. vika*. Given the species has been recorded only in primary forests below 250 m elevation, and Dokoso tribal land represents the last major area of this habitat, logging of Dokoso would undoubtedly lead to the extinction of *U. vika*. This would contradict the commitment made by the Solomon Islands National Government as a signatory to the Convention on Biological Diversity, specifically: “to prevent the extinction of known threatened species and improve and sustain their conservation status, particularly of those most in decline” (United Nations, 1993).

## AUTHOR CONTRIBUTIONS

**Tyrone H. Lavery:** Conceptualization (equal); data curation (equal); formal analysis (equal); funding acquisition (equal); investigation (equal); methodology (equal); project administration (equal); writing – original draft (lead); writing – review and editing (lead). **Adrian Holland:** Conceptualization (equal); investigation (equal); methodology (equal); writing – original draft (supporting); writing – review and editing (supporting). **Nixon Jino:** Conceptualization (equal); investigation (equal); methodology (equal); project administration (equal); writing – original draft (supporting); writing – review and editing (supporting). **Atuna Judge:** Investigation (equal); methodology (equal); project administration (equal); writing – original draft (supporting); writing – review and editing (supporting). **Hikuna Judge:** Investigation (equal); methodology (equal); project administration (equal); writing – original draft (supporting); writing – review and editing (supporting). **Pandakai Onga:** Investigation (equal); methodology (equal); project administration (equal); writing – original draft (supporting); writing – review and editing (supporting). **Kevin Sese:** Conceptualization (equal); data curation (equal); methodology (equal); project administration (equal); writing – original draft (equal); writing – review and editing (equal).

## CONFLICT OF INTEREST STATEMENT

Dr Tyrone Lavery provided expert opinion for an appeal before the Solomon Islands Advisory Committee concerning the issuance of development consent for logging operations inside Dokoso customary land, southern Vangunu.

## Data Availability

Examples of key data collected during this study (images of *Uromys vika*) are presented here in this published article. Accurate GPS localities for camera traps have been withheld and are available from the corresponding author upon reasonable request.
